# Caveolin as a Universal Target in Dermatology

**DOI:** 10.3390/ijms21010080

**Published:** 2019-12-20

**Authors:** Ilja L. Kruglikov, Philipp E. Scherer

**Affiliations:** 1 Scientific Department, Wellcomet GmbH, 76229 Karlsruhe, Germany; I.Kruglikov@wellcomet.de; 2 Touchstone Diabetes Center, Department of Internal Medicine, University of Texas Southwestern Medical Center, Dallas, TX 75390-8549, USA

**Keywords:** Caveolin-1, dermatology, psoriasis, wound healing, scarring, skin aging, pathophysiology, target

## Abstract

Caveolin-1 is strongly expressed in different dermal and subdermal cells and physically interacts with signaling molecules and receptors, among them with transforming growth factor beta (TGF-β), matrix metalloproteinases, heat shock proteins, toll-like and glucocorticoid receptors. It should therefore be heavily involved in the regulation of cellular signaling in various hyperproliferative and inflammatory skin conditions. We provide an overview of the role of the caveolin-1 expression in different hyperproliferative and inflammatory skin diseases and discuss its possible active involvement in the therapeutic effects of different well-known drugs widely applied in dermatology. We also discuss the possible role of caveolin expression in development of the drug resistance in dermatology. Caveolin-1 is not only an important pathophysiological factor in different hyperproliferative and inflammatory dermatological conditions, but can also serve as a target for their treatment. Targeted regulation of caveolin is likely to serve as a new treatment strategy in dermatology.

## 1. Introduction

The plasma membrane of eukaryotic cells has a spatially heterogeneous structure containing cholesterol- and sphingolipid-enriched lipid rafts that can appear in the form of planar structures or plasma membrane invaginations, known as caveolae. Caveolae are distinct from other plasma membrane lipid rafts, primarily due to the presence of caveolins and their interaction with cholesterol. Caveolin-1 (Cav-1) is the principal structural component of caveolae, which interacts with the cell cytoskeleton on the inside and with extracellular matrix (ECM) on the outside of the cell [1] and which is often considered as a master regulator of cellular signaling. Cav-1 indeed physically interacts with different signaling molecules and receptors, among them with transforming growth factor beta (TGF-β). In addition, direct physical interactions have been established between Cav-1 and some matrix metalloproteinases, heat shock proteins, toll-like and glucocorticoid receptors. Cav-1 is causally involved in processes such as collagen production, interaction of hyaluronan with plasma membranes, and the regulation of the autophagy [2,3,4,5]. Additionally, Cav-1 exists not only intracellularly, but it can be transported within the tissue, and even between adjacent tissues, via mechanisms involving exosomal exchange [6] providing a long-range distribution of this protein in the skin.

Recent findings demonstrate the involvement of Cav-1 in different hyperproliferative and inflammatory skin conditions, making this protein an important pathophysiological factor in psoriasis [2], hypertrophic scarring [3], and acne [4]. Cav-1 is also involved in wound healing, where it demonstrates a “biphasic” behavior: whereas cutaneous wound healing was found to be significantly slower in caveolin-1 knock out (Cav-1 KO) mice compared to their wild counterparts [7], the healing time in corneal epithelium linearly increased with increasing Cav-1 expression with aging [8]. Very recent data suggests that the induced overexpression of Cav-1 in epidermal stem cells significantly promotes their proliferative ability and accelerates wound closure in burn wounds [9]. These and some other skin conditions are characterized by impaired expression of Cav-1, leading to deregulated cellular signaling, strongly modified collagen production and hyaluronan accumulation, as well as to an abnormal mechanical behavior of cells.

On the other hand, Cav-1 is overexpressed in aging skin [5], which is generally connected with a reduction in the number of immature cells, development of cellular senescence, and strong modulation of the ECM structure. Delayed wound healing in diabetes is connected with substantial overexpression of Cav-1 and induction of cellular senescence in dermal fibroblasts, whereas suppression of Cav-1 ameliorated both premature senescence and impaired wound healing [10]. This clearly demonstrates that both reduced expression and overexpression of Cav-1 can lead to the emergence of pathological skin conditions.

Both stimulation and reduction of Cav-1 expression levels can be achieved with the same methods, by modulating their strength and time of application [2,3,4]. Cav-1 expression is not only a pathophysiological factor in different skin conditions, but can also serve as a target for their improvement. This opens new perspectives in dermatology and aesthetic medicine. Here we present a critical discussion of recent results in this field.

## 2. Caveolin-1 (Cav-1) in the Hippo Pathway

Regulation of caveolin expression is connected with the Hippo pathway which plays an important role in tissue growth restriction and cell migration. This pathway is strongly involved in proliferation and differentiation of stem cells, thus being an important regulatory instrument in skin homeostasis [11]. Severe disturbances of this pathway were found in different hyperproliferative and inflammatory skin diseases as well as in neoplasia. Expression of Yes-associated protein (YAP) and transcriptional coactivator with PDZ-binding motif (TAZ), the major transcriptional mediators of the Hippo pathway, was reported to be significantly increased in clinical psoriatic specimens as well as in imiquimod-based mouse model of psoriasis [12]. Its expression was also strongly enhanced in the injured dermis two days after cutaneous wounding and these elevated levels were found in the whole wound area by day seven, whereas a knockdown of YAP/TAZ markedly reduces the expression of TGF-β and delayed wound closure [13]. YAP was also found to be strongly overexpressed in skin lesions of lichen planus [14].

The Hippo pathway can be regulated by a wide range of signals, including chemical ligands and mechanical stimuli [15]. Its activity is critical for the formation of caveolae in mammalian cells, and Cav-1 is an important negative regulator of YAP/TAZ [16]. Moreover, the lack of caveolae causes hyperactivation of YAP/TAZ. On the other hand, these Hippo mediators are involved in epithelial proliferation [17], and YAP promotes abnormal proliferation of psoriatic keratinocytes [12]. Cav-1 expression regulates YAP activity in response to changes in ECM stiffness [18].

These results collectively demonstrate that Cav-1 expression is potently involved in the regulation of cell proliferation and differentiation, and thus contributes to the appearance of different hyperproliferative skin conditions.

## 3. Role of Cholesterol in Caveolae Stability and Cav-1 Expression

The plasma membrane of human fibroblasts contains 90% of the total cellular cholesterol, which is necessary for the assembly of the caveolin complex from its subunits and for the stabilization of the caveolae in the plasma membrane [19]. Cholesterol efficiently regulates the membrane fluidity in a concentration-dependent manner [20], significantly increasing membrane thickness, the depth of Cav-1 insertion into membranes, and the resistance of plasma membranes to disruption under mechanical stress [21].

The reduction in cellular cholesterol by application of U18666A (an inhibitor of cholesterol trafficking), dramatically increased the level of free, rapidly diffusing Cav-1 in the plasma membrane at the expense of caveolae [19]. Application of methyl-β-cyclodextrin (MβCD), which is a cholesterol-depleting agent disrupting caveolae and demonstrating skin-specific anti-Cav-1 activity, strongly reduces Cav-1 expression in murine and human skin cells [22] and provides redistribution of Cav-1 from the periphery into the internal regions of the plasma membrane [23]. Treatment with MβCD also led to a reduction of the secretion of epidermal lamellar bodies [24], impairing the desquamation of keratinocytes. Under quasi-physiological conditions, interaction between cholesterol and Cav-1 in the plasma membrane appears to be reciprocal: whereas cholesterol enrichment/depletion increases/decreases Cav-1 expression, a knockdown of Cav-1 provides a remarkable decrease in the membrane cholesterol content in human mesenchymal stem cells [25]. Deoxycholic acid (DCA), which is widely applied in injection lipolysis, causes redistribution of cholesterol providing its aggregation into large patches and an increase of its light fractions; simultaneously, DCA decreases the Cav-1 content in the plasma membrane, initiating its internalization [26]. Of note, application of MβCD distinctly ameliorates the effects of DCA.

Altogether, treatment methods modulating cholesterol content in plasma membrane have a significant impact on Cav-1 expression.

## 4. Some Reasons for Cav-1 Deficiency

Cav-1 deficiency in hyperproliferative and inflammatory skin conditions can have different reasons. Here we will shortly discuss only some of them.

### 4.1. Prevalence of Immature Cells in the Tissue

Reduced Cav-1 content in the tissue can reflect the shift in the ratio of immature-to-mature cells to the immature pool. Indeed, Cav-1 is differentially expressed in immature and mature cells; for example, Cav-1 expression dramatically increases during adipogenesis demonstrating a much higher levels in mature adipocytes than in adipogenic precursors [27,28]. Cav-1 deficient mice have an increased mammary stem cell population [29], which reflects the impaired ability of these cells to differentiate.

Application of MβCD revealed strong correlation between lipid rafts and differentiation ability of human mesenchymal stem cells [23]. Cav-1 expression in epidermal stem cells plays a critical role in their proliferative ability and induced expression of Cav-1 in these cells provides significantly accelerated wound closure [9]. Transient downregulation of Cav-1 expression may be required for the transition between immature and mature states [30]. Of note, the ratio of immature-to-mature cells in the tissue is not necessarily spatially constant. For example, adipose stromal cells can be redistributed in the tissue through chemotaxis [31].

Cells with a low Cav-1 content fail to adjust their stiffness to the mechanical characteristics of the extracellular matrix (ECM). This makes the proliferation and differentiation of such cells essentially independent of the ECM producing conditions normally leading to a hyperplasia. Specific re-expression of Cav-1 was able to restore the mechanical sensitivity of cells to ECM [32]. This communication plays an important role in cell commitment: mesenchymal stem cells seeded on substrates having differential elastic moduli differentiate into specific cell types [33].

A shift in the ratio of immature-to-mature cells in the tissue can have pathological and physiological reasons, and can be drug-induced as well. Pathologically, the proliferative and differentiative activity of epithelial stem cells significantly increases after cutaneous wounding, demonstrating a specific spatiotemporal distribution [34,35]. As these cells have high migrative activity [36], their expression of adhesion receptors is strongly reduced. It was indeed reported that reduced expression of integrins (the major class of the cell substrate receptors) in human mesenchymal stem cells is related to reduced expression of Cav-1 [25].

Interestingly, the undifferentiated myogenic precursor (satellite) cells, which quickly proliferate and fuse with damaged fibres after muscle injury, also demonstrate a characteristic temporal modulation of Cav-1 expression [37]. Under physiological conditions, satellite cells are quiescent— they overexpress Cav-1 inducing cell cycle arrest, but the downregulation of Cav-1 is able to induce cycling in these cells. Satellite cells begin to migrate to the wounded area shortly after injury, and this migration is accompanied by a transient downregulation of Cav-1. Cav-1 expression returned to its basal level three days after injury, which temporally correlates with differentiation of myogenic precursors into myotubes. At the same time, myogenic precursors in transgenic mice overexpressing Cav-1 were unable to rapidly downregulate their Cav-1 levels upon injury and could not migrate to the wounded area, which resulted in impaired muscle regeneration in these animals [37]. Such temporal Cav-1 regulation is of general biological importance and can be realized also in injured skin, although with other players.

### 4.2. Processes of De- and Re-Differentiation in Dermal White Adipose Tissue (dWAT)

Dermal white adipose tissue (dWAT) is the special fat depot located in the superficial hypodermis and around the lower parts of the pilosebaceous units. This depot demonstrates remarkable volume oscillations during hair follicle (HF) cycling [38,39].

As we discovered very recently, these variations of the dWAT volume are mainly connected with repeated de- and re-differentiation processes, which affect almost 95% of the adipocytes from dWAT [40]. De-differentiation of mature dermal adipocytes into adipocyte-derived preadipocytes (ADPs) in catagen provides a dramatic reduction of the cell volumes in dWAT [39]. During this process, dWAT serves as a local source for fatty acids [40], which can be transported to the dermis. The inverse process—re-differentiation of ADPs into mature adipocytes—takes place during anagen and demands a significant influx of fatty acids. Consequently, the dermis experiences oscillations of fatty acid loading during HF cycle. Since Cav-1 is strongly involved both in uptake and efflux of lipids [41,42], these oscillations must correlate with the local Cav-1 content in the areas adjacent to the pilosebaceous units. This effect must make some pathological skin conditions HF-cycle-dependent [4]. Indeed, initiation of the acne lesions is connected with the catagen/telogen phases of the HF cycle [43]. Similarly, the content of telogen and catagen hairs are significantly higher in psoriatic lesions compared to uninvolved skin [44,45].

As demonstrated in [40], ADPs can be alternatively transdifferentiated into myofibroblasts, which typically have significantly lower levels of Cav-1 expression compared to fibroblasts and the differentiation into myofibroblasts is enhanced under Cav-1 deficiency [3]. The detailed signaling pathways mediating the transition from the cyclic de-/re-differentiation of dermal adipocytes to their transdifferentiation into myofibroblasts are still to be elucidated, but the involvement of TGF-β is very likely.

### 4.3. Modification of Cholesterol in Plasma Membrane

Peroxidation of plasma membrane lipids leads to the formation of “crystalline” cholesterol domains, which are known to be involved in inflammation. Glucose treatment of the model membranes containing cholesterol at physiologic levels provided a dose-dependent formation of lipid hydroperoxide and crystalline cholesterol domains [46]. This effect was dependent on the initial cholesterol level in the plasma membrane. Hyperglycemia also causes a significant reduction of the number and size of caveolae in macrophages [47].

The content of the oxidized low-density lipoprotein (oxLDL) is known to be strongly modified in different skin efflorescences. For example, significant accumulation of oxLDL was detected in the upper epidermis of the lesional, but not of the uninvolved psoriatic skin [48]. Analysis in vitro demonstrated that the induction of oxLDL increases the migration of keratinocytes and the expression of lectin-type oxLDL receptor 1, which is the main receptor for oxLDL in endothelial cells and whose overexpression was recently connected with psoriasis [49]. Endothelial cells exposed to 10 mg/mL oxLDL for 1 h displayed a dramatic depletion of cholesterol and more than 90% depletion of Cav-1 content in caveolae [50]. At the same time, application of oxLDL in lower doses (20–120 µg/mL for 12–48 h) demonstrated rapid and significant (up to 3-fold) upregulation of Cav-1 expression [51]. These findings demonstrate that the low-dose oxLDL exposure induces Cav-1 expression and promotes the appearance of caveolae, whereas its high-dose exposure causes Cav-1 depletion. As we discuss later, such a biphasic reaction of Cav-1 is typical for different drugs and physical forces.

Whereas the reduction of cholesterol after application of statins was formally confirmed only for plasma membranes of erythrocytes and platelets [52], it is likely that this reaction can also be seen in other cell types, including epithelial and endothelial cells. Statins were reported to be effective for the treatment of psoriasis and atopic dermatitis, but can also induce psoriasis-like conditions [53]. This means that under different conditions, statins can suppress or induce expression of Cav-1. Indeed, whereas statins are generally believed to reduce the abundance of Cav-1 [54,55,56], lovastatin and pravastatin induce Cav-1 in macrophages [57].

### 4.4. Non-Physiological Autophagy

Silencing of Cav-1 in keratinocytes in vitro caused an increased expression of different cytokines/chemokines, such as IL-6, CXCL8, and CXCL9, whereas the application of a Cav-1 scaffolding domain peptide in a murine skin model of psoriasis effectively reduces their expression [58]. On the other hand, the stimulation of keratinocytes with TNF-α effectively reduces the Cav-1 mRNA expression [58], which led to the conclusion that skin inflammation may suppress Cav-1 expression.

TNF-α induces autophagy in different types of cells, and this process was assumed to protect cells from apoptosis [59,60,61]. Autophagy and cellular cholesterol demonstrate a cross talk: keratinocytes stimulated with IL-17A have inhibited autophagy accompanied by enhanced cholesterol levels in these cells [62,63]. Since the application of MβCD significantly reduces the effects of IL-17A, it can be concluded that the intracellular accumulation of cholesterol must be essential for IL-17A signaling [62].

Under physiological conditions, Cav-1 is co-localized with different autophagy proteins and even acts as a critical determinant of autophagy [64]. However, when autophagy is prolonged or strongly overstimulated, Cav-1 will be degraded [65,66,67], which can contribute to a local Cav-1 deficiency in the tissue. The autophagic degradation of Cav-1 can, among others, be induced by palmitic acid (PA), which is a typical component of sebum [68] and which can be also released by enlarged adipocytes [69]. PA-induced autophagy is Cav-1-independent, but the degradation of Cav-1 is responsible for the development of inflammation, and chronic high-fat diet induces autophagy and Cav-1 degradation [68]. Moreover, induced expression of Cav-1 was shown to attenuate these effects. Elevated levels of PA induce enhanced uptake of oxLDL in macrophages [70], which demonstrates an interconnection between different processes leading to Cav-1 deficiency.

## 5. Cav-1 as a Target in a Variety of Pathological Cutaneous Conditions

A number of observations indicate that Cav-1 is not only a pathophysiological factor but can also serve as a target in various hyperproliferative and inflammatory skin conditions, among them in psoriasis, acne, atopic dermatitis, cutaneous fibrosis, wound healing, and skin aging. For example, decreased expression of Cav-1 is involved in pathogenesis of psoriasiform dermatitis in a murine model, and enhanced expression of Cav-1 through administration of a Cav-1 scaffolding domain peptide, which is a functional mimetic of Cav-1, provides substantial improvement of this skin condition [58]. On the one hand, Cav-1 deficiency promotes both basal and inducible autophagy, enhancing lysosomal function and autophagosome-lysosome interaction [71]; on the other hand, re-expression of Cav-1 suppresses autophagy [68,72]. This supports a potent regulatory role of caveolin in these processes.

Induced expression of Cav-1 promotes proliferation of epidermal stem cells. Such stimulation significantly improves the healing of burn wounds [9]. Application of curcumin prompts an upregulation of Cav-1 expression in these cells. In contrast, genetic ablation of Cav-1 almost completely abrogates the effects of curcumin on cell proliferation and wound closure [73]. This mechanism may be involved in skin improvements observed after topical application of curcumin in different inflammatory skin disorders, including psoriasis and cutaneous wounds. In contrast, delayed wound healing in diabetes is connected with overexpression of Cav-1, induced by oxidative stress, which leads to the development of the cellular senescence [10]. Causal involvement of Cav-1 is confirmed via a knockdown of Cav-1, a condition that ameliorates both premature senescence and impaired wound healing.

The involvement of Cav-1 in wound healing consequently leads to a paradoxical situation. Normally, mechanical and burn wounds demonstrate a reduction of Cav-1 levels shortly after injury caused by activation of proliferation and migration of epithelial stem cells into the wound area. Such a suppression of Cav-1 should recover after several days in the case of regenerative wound healing. However, when Cav-1 suppression is too strong or too long, the recovery of Cav-1 levels can be impaired, which can lead to delayed wound healing and higher risk of hypertrophic or keloid scarring. On the other hand, diabetic wounds generally demonstrate impaired healing, which can be connected with the fact that the cells overexpress Cav-1 and are not able to provide its quick downregulation. Paradoxically, such pathophysiology predicts reduced scarring after healing of diabetic wounds relative to burns. Correspondingly, differential treatment strategies for burns and diabetic wounds may be indicated due to the differences in Cav-1 expression.

Cav-1 expression also demonstrates correlations with aging, both in vitro and in vivo. Oxidative stress upregulates Cav-1 expression causing premature cellular senescence [74]. Very recently, it was reported that UV-B radiation in small doses significantly enhances expression of Cav-1 in melanocytes and keratinocytes in vitro [75]. Development of the cellular senescence in vitro can be reversed by specific reduction of Cav-1 levels [76], indicating that excess of Cav-1 may be causally connected with the aging process. These findings are in the line with reported anti-aging effect of MβCD on the skin [22]. Collectively, these results support the central role of Cav-1 in pathogenesis of different hyperproliferative and inflammatory skin disorders, as well as its possible role as a target for their regulation.

## 6. Approaches to Regulate Cav-1 Expression Levels

Under physiological conditions, caveolae in the plasma membrane are stable: the half-life of Cav-1 is estimated to be about 24–36 h [19,21]. The manipulation of the Cav-1 content can be induced by application of different physical stimuli (mechanical, hypo-osmotic, and thermal stress) and pharmacological agents.

The mechanical modulation of Cav-1 demonstrates a biphasic, dose-dependent behavior, similar to what takes place during lipid oxidation: low-level stimulation normally provides an enhanced Cav-1 expression, whereas strong stimulation causes Cav-1 degradation [77]. The modulation of caveolar density in plasma membranes is tightly connected with a redistribution and modification of the actin cytoskeleton. Reaction of the cellular cytoskeleton strongly depends on the strength of the mechanical forces applied and can be either stiffening or softening. Stiffening of human skin fibroblasts in vivo was shown to be typical in aging [78]. The application of MβCD disrupting caveolae and reducing Cav-1 provides a softening of the cellular membranes [32]. Thus, the processes of cellular stiffening and softening is directly connected with expression of Cav-1.

Physical changes caused by large mechanical strains of about 10% at physiological frequencies of about 1 Hz are closely analogous to those caused by very small strains at ultrasonic frequencies of 1 MHz [79]. Application of ultrasound waves of higher intensities and higher frequencies provides a stronger softening of the cytoskeleton [80]. Ultrasound also causes caveolar internalization of glutathione-s-transferase in a dose-dependent manner [81]. Of note, ultrasound was reported to be able to reverse the healing delays in diabetes and aging through activation of the Ras-related C3 botulinum toxin substrate (Rac1) [82]. Rac1 binds Cav-1 and promotes its accumulation in peripheral adhesions; on the other hand, Cav-1 controls Rac1 expression regulating its ubiquitylation and degradation [83].

Recent analysis of expression profiles of ultrasound-induced genes revealed that the *CAV1* gene was indeed strongly upregulated [84]. Application of ultrasound waves also significantly enhanced Cav-1 expression in HEp-2 cells and endothelial cells in a time and dose-dependent manner [85,86]. All this makes the very high frequency ultrasound (VHF-US) to an interesting modality in manipulation of Cav-1 expression [2].

Mild hyperthermia with supra-physiological temperatures is also able to stimulate the Cav-1 expression in different types of cells [87,88], whereas strong hyperthermia leads to degradation of Cav-1 and its internalization [89]. This supports the general character of biphasic responses of Cav-1 to external factors. VHF-US ultrasound with frequencies over 10 MHz was shown to induce supra-physiological temperatures, thus providing the frequency-dependent thermo-mechanical stress in different skin layers and in subcutis [90]. Of note, both VHF-US and mild hyperthermia were successfully applied for the treatment of psoriasis (see discussion in [2]).

The effect of different drugs, which are applied for the treatment of various hyperproliferative and inflammatory skin disorders, can be also directly or indirectly connected with the manipulation of Cav-1 expression. Information for some of them is summarized in Table 1.

## 7. Role of Cav-1 in Drug Resistance

A proper modulation of Cav-1 content can significantly influence the cell sensitivity to different cytostatic agents. Induced overexpression of Cav-1 in drug resistant cells, which are characterized by low levels of endogenous Cav-1, reduced their resistance to doxorubicin and cisplatin by 97% and 64%, respectively [101]. Methotrexate is another cytotoxic drug demonstrating effectiveness in some dermatological conditions, such as psoriasis and atopic dermatitis [63]. Whereas the methotrexate-resistant cancer cells demonstrate strong overexpression of Cav-1, which is typical in advanced cancer stages, application of siRNA against Cav-1 effectively decreased this resistance [102]. Downregulation of Cav-1 also increased the sensitivity of cancer cells to 5-flourouracil (5-FU) [103], which is also often applied for the treatment of hypertrophic scars.

These results demonstrate that proper modulation of Cav-1 expression could be a supportive therapy in application of cytotoxic drugs. Modulation of Cav-1 as a supportive therapy in cancer treatment is clearly recognized and intensively discussed during the recent past [72,103,104]. At the same time, drug resistance in treatment of dermatological conditions was until now not directly connected with Cav-1 expression.

One of these drugs is the all-trans-retinoic acid (ATRA), which is successfully applied in cancer therapy as well as for an array of different dermatological conditions, including acne and skin aging. The impact of ATRA applications is generally limited by rapid emergence of acquired resistance [105]. ATRA redistributes Cav-1, increasing its membrane fraction and reducing cytosolic content [91]. On the other hand, ATRA significantly reduces Cav-1 in endothelial cells that overexpress Cav-1 [92], which clearly demonstrates that the regulatory role of this drug is dependent on the expression levels of Cav-1. An additional important effect of ATRA is the upregulation of the nuclear transcription factor FOXO1 [93], whose overexpression induces Cav-1 [94]. These results indicate that acquired resistance to ATRA can be connected with an impaired expression of Cav-1 in the tissue. Since the main hyperproliferative and inflammatory conditions in dermatology are connected with impaired Cav-1 expression in the tissue, proper manipulation of Cav-1 can not only provide a direct impact on skin improvements, but can also serve as a supportive therapy in the context of applications of drugs in dermatology. This hypothesis should be carefully examined in future research.

## 8. Conclusions

Cav-1 expression is not only an important pathophysiological factor in different hyperproliferative and inflammatory skin conditions, but can also serve a target for their treatment. Moreover, manipulation of Cav-1 expression can be an important supportive therapy increasing sensitivity of skin cells to different drugs. Whereas induced stable expression of Cav-1 improves skin appearance in psoriasis, burn wounds, and scarring, the suppression of Cav-1 can be considered as a treatment objective in aging skin. Since one and the same treatment depending on its intensity can induce either expression or suppression of Cav-1, optimization and personalization of the treatment regime must be of special importance in these treatment strategies.

## Figures and Tables

**Table 1:** Some studies demonstrating involvement of Caveolin-1 (Cav-1) in the effect of different drugs in dermatology.

No	Drug	Effect on Cav-1	Reference
1	Retinoids	All-trans retinoic acid strongly increased Cav-1 localization in plasma membrane. Retinoic acid significantly reduced Cav-1 in endothelial cells initially having an increased Cav-1 level. Retinoic acid can induce Cav-1 in Cav-1 deficient cells through upregulation of Forkhead Box O1 (FOXO1).	[91,92,93,94]
2	Corticosteroids	Dexamethasone induced Cav-1 expression in endothelial cells. Dexamethasone induced Cav-1 expression in alveolar epithelial cells.	[95,96]
3	Curcumin	Curcumin significantly upregulated Cav-1 expression in epidermal stem cells. Genetic ablation of Cav-1 abrogated this effect. Curcumin inhibited cholesterol accumulation through stimulation of Cav-1 expression.	[73,97]
4	Statins	Rosuvastatin and atorvastatin reduced Cav-1 in apoE^−/−^ mice in vivo. Simvastatin reduced Cav-1 in myocardium by 84%. Lovastatin and pravastatin strongly induced Cav-1 in macrophages.	[54,55,57]
5	Bleomycin (BLM)	BLM decreased Cav-1 expression in epithelial cells. Cav-1 is strongly reduced in BLM-induced lung fibrosis	[98,99]
6	Fluorouracil (5-FU)	5-FU strongly upregulated Cav-1 in breast cancer cells both in vitro and in vivo.	[100]
